# Forebrain Glucocorticoid Receptor Overexpression Alters Behavioral Encoding of Hippocampal CA1 Pyramidal Cells in Mice

**DOI:** 10.1523/ENEURO.0126-22.2022

**Published:** 2022-12-05

**Authors:** Swapnil Gavade, Qiang Wei, Colin Johnston, Savannah Kounelis-Wuillaume, Klaudia Laborc, Salisha Baranwal, Huda Akil, Joanna L. Spencer-Segal

**Affiliations:** 1 Michigan Neuroscience Institute, University of Michigan, Ann Arbor, MI; 2 Department of Internal Medicine, University of Michigan Medical School, Ann Arbor, MI

**Keywords:** CA1, glucocorticoid, hippocampus, miniscope, open field

## Abstract

Glucocorticoid signaling influences hippocampal-dependent behavior and vulnerability to stress-related neuropsychiatric disorders. In mice, lifelong overexpression of glucocorticoid receptor (GR) in forebrain excitatory neurons altered exploratory behavior, cognition, and dorsal hippocampal gene expression in adulthood, but whether GR overexpression alters the information encoded by hippocampal neurons is not known. We performed *in vivo* microendoscopic calcium imaging of 1359 dorsal CA1 pyramidal cells in freely behaving male and female wild-type (WT) and GR-overexpressing (GRov) mice during exploration of a novel open field, where most CA1 neurons are expected to respond to center location and mobility. Most neurons showed sensitivity to center location and/or mobility based on single-neuron calcium amplitude and event rate, but these sensitivity patterns differed between genotypes. GRov neurons were more likely than WT neurons to display center sensitivity and less likely to display mobility sensitivity. More than one-third of these responsive GRov neurons were sensitive only to center location and not mobility, while uniquely center-sensitive neurons were rare in WT. Most center-sensitive neurons exhibited anticipatory activity, suggesting they could drive behavior. We conclude that, compared with wild-type, dorsal CA1 pyramidal cells in GRov mice preferentially respond to center location rather than mobility in a novel open field. Such changes in the information encoded by individual hippocampal neurons in an aversive environment could underlie changes in stress vulnerability because of genetic or epigenetic variations in glucocorticoid receptor signaling.

## Significance Statement

Glucocorticoids alter hippocampal-dependent behaviors and vulnerability to stress-related disorders. Here, we find that increased sensitivity to glucocorticoid via lifelong overexpression of glucocorticoid receptor in forebrain neurons (GRov) changes the information encoded by individual hippocampal neurons in a mildly aversive environment, the novel open field. GRov neurons showed heightened sensitivity to center location and lower sensitivity to mobility. These changes in hippocampal neuronal sensitivity could underlie the differences in stress vulnerability in humans with genetic and epigenetic differences in glucocorticoid receptor signaling or excess glucocorticoid exposure during development.

## Introduction

Glucocorticoid receptors (GRs) are found in neurons throughout the brain, where they contribute to baseline and stress-related behavior (McEwen et al., 2015; McEwen and Akil, 2020). In particular, the hippocampus is highly sensitive to glucocorticoids, and acute, chronic, and developmental glucocorticoid exposures influence hippocampal-dependent behaviors including affect and cognition (McEwen and Gianaros, 2011; Moisiadis and Matthews, 2014a,b). In humans, genetic and epigenetic changes in the GR alter the risk for hippocampal-dependent disorders such as posttraumatic stress disorder and cognitive impairment (Koper et al., 2014; Palma-Gudiel et al., 2015; Yehuda et al., 2015; Kang et al., 2018). Despite this clear, cross-species link between glucocorticoid receptor signaling and hippocampal-dependent behaviors, how changes in glucocorticoid receptor signaling alter hippocampal neuronal function is not known.

To determine how neuronal glucocorticoid signaling across the lifespan alters brain function and behavior, mice were previously generated with constitutive and conditional glucocorticoid receptor overexpression in excitatory forebrain neurons (GRov mice; Wei et al., 2004, 2012). GRov mice display altered hippocampal-dependent behaviors, including decreased exploration of a mildly aversive environment (commonly interpreted as anxiety-like behavior) and impaired spatial memory without differences in basal or mild stress-induced corticosterone levels (Wei et al., 2004, 2007). The anxiety-like behavioral phenotype of the lifelong GRov mouse in adulthood was recapitulated by conditional GR overexpression during the first three weeks of life but not after weaning, suggesting that the phenotype is developmentally programmed (Wei et al., 2012). GRov mice also show profound lifelong changes in gene expression in the dorsal hippocampus (Wei et al., 2012). This evidence suggests that early life GR overexpression in forebrain excitatory neurons alters hippocampal function in adulthood.

The goal of this study was to determine how lifelong GR overexpression alters the function of hippocampal neurons in adulthood. To do this, we used *in vivo* calcium imaging to record CA1 pyramidal neuron activity from male and female wild-type (WT) and GRov mice during free exploration of a brightly lit, novel open field. Dorsal CA1 was chosen because of its importance for exploratory and cognitive behavior and its sensitivity to GRov as evidenced by gene expression analysis. Dorsal CA1 neurons are expected to encode information about the environment and behavior during open field exploration, including center location and speed (Hainmueller and Bartos, 2018; Jimenez et al., 2018; Iwase et al., 2020). After recording the activity of 1359 neurons, we identified center and mobility sensitivity at the single-neuron level and compared the proportion of sensitive neurons between genotypes to uncover genotype differences in CA1 neuron function.

## Materials and Methods

### Subjects

GRov mice were previously generated using the CamKIIα promoter to direct expression of mouse GR, resulting in GRov overexpression primarily in forebrain glutamatergic neurons (Wei et al., 2004). The GRov line was established by breeding founders and their progeny to C57Bl6/J mice, and the line was maintained by breeding heterozygotes. Homozygote GRov and WT male and female littermates aged three to six months were used for these studies. Mice were maintained on a 14/10 h light/dark cycle with imaging sessions performed during the light phase. All animal procedures were conducted in accordance with the University of Michigan animal care committee’s regulations.

### *In vivo* microendoscopic calcium imaging

**Figure 1.**
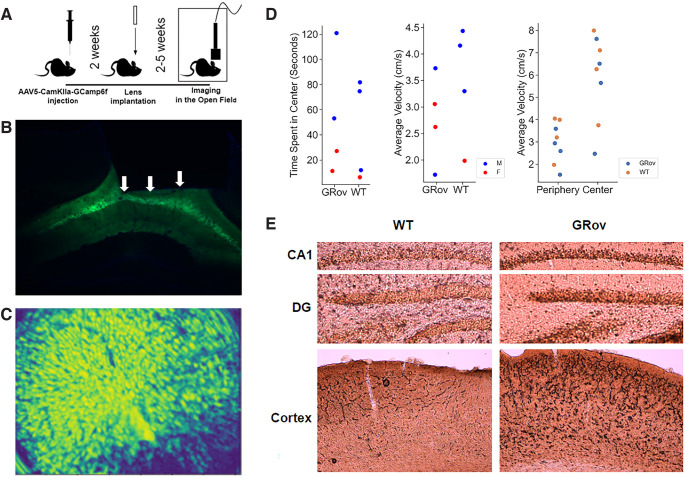
***A***, Schematic showing AAV5-CamKlla-Gcamp6f injection, lens implantation, and calcium imaging in open field arena steps for the experiment. ***B***, Histology demonstrating GCamp6f expression in dorsal CA1 pyramidal cells. White arrows delineate the lower lens border. ***C***, Representative frame from calcium imaging recording after applying spatial filtering. ***D***, Behavior of each mouse in the experiment including total time spent in the center, velocity during the entire trial, and velocity (cm/s) in the periphery and center**. *E***, Increased immunolabeling for GR in the CA1, dentate gyrus, and cortex of representative male WT and GRov mice.

*In vivo* calcium imaging in freely behaving mice was performed using the nVista 2.0 miniature endoscope (Inscopix) following the procedures described in detail elsewhere (Resendez et al., 2016). Young adult male and female GRov and WT mice underwent injection of AAV5-CamKIIa-GCamp6f (Addgene; 300 nl diluted 1:5 in artificial CSF AP −2.05, ML 1.75 from bregma; DZ −1.3 from skull) and implantation of a 4 × 1 mm GRIN lens (at AP −1.95, ML 1.6 from bregma, DZ −1.55 from skull) over the dorsal hippocampal CA1 pyramidal cell layer (Fig. 1*A*,*B*).

For recordings, mice were allowed to explore a large (72 × 72 cm) brightly lit (200 lux) open field for 10 min. Behavior was recorded and analyzed using Noldus Ethovision XT (version 12) and synchronized with calcium imaging using TTL pulses with a Noldus I/O Box. At least 3 days after the completion of behavioral testing, mice were perfused with PBS followed by 4% paraformaldehyde and brains were postfixed in paraformaldehyde for 24 h before removal of the lens. 40-μm sections were cut on a cryostat and used to verify GCamp6f expression in dorsal CA1 pyramidal cells and accurate placement of the lens over the dorsal CA1 region.

### Data processing

Downsampling (by two in space and time), cropping and trimming of videos were performed using Inscopix Data Processing Software (IDPS 1.6.0). CaImAn, an open-source tool for scalable calcium imaging data analysis (Zhou et al., 2018), was used to perform background subtraction, cell identification, and Δf/F trace extraction on collected calcium imaging data using the Python toolbox. Manual curation of the identified cells was performed by visual inspection to ensure that the final accepted cells were unique and exhibited typical calcium dynamics, with transients characterized by fast rise times and exponential decays. Event detection through deconvolution was performed using the Online Active Set method to Infer Spikes method (Friedrich et al., 2017). The experimenter who processed the calcium imaging data were blind to treatment groups until the neurons were segregated into groups for genotype comparisons.

To determine the behavior sensitivity of hippocampal neurons, calcium activity was aligned with behavior including location (center and periphery) and mobility, which was designated high or low based on velocity ≥5 or <5 cm/s, respectively. The center area was a 52-cm square defined in the center of the 72-cm square arena, and outside the center was defined as the periphery. Calcium amplitude was shuffled for each animal to generate a shuffled data distribution, a “null” distribution where the calcium activity will not be sensitive to any behavior. For each neuron, a ratio was calculated for the average calcium amplitude in the center/periphery, and high/low mobility. Thresholds for behavioral sensitivity were defined as cells with a ratio lower than the first percentile or higher than the 99th percentile, based on the shuffled data distribution within genotype (Fig. 1*E*). These cells were considered mobility-sensitive or center-sensitive and represent cells that selectively increase or decrease their calcium activity when the mouse increases velocity or enters the center of the open field (high and low mobility cells, and center and periphery cells). Whether calcium activity anticipated a behavioral change was determined by comparing calcium activity during the 1-s period before the mouse entered the center relative to the average calcium activity in the periphery.

### Immunohistochemistry

Brain sections were washed in Tris-buffered saline (TBS) and 0.5% bovine serum albumin (BSA) for 30 min each, then incubated overnight at room temperature in 1:400 primary antibody (rabbit anti-GR from Cell Signaling Technology, D8H2 #3660). The next day, they were rinsed in TBS for 45 min and incubated in 1:200 secondary antibody (biotinylated goat anti-rabbit IgG, Vector Laboratories #BA-1000) for 30 min at room temperature. The sections were then washed in TBS for 45 min and incubated in avidin-biotin-peroxidase complex (ABC) solution (Vector Laboratories) for 30 min, followed by another 40-min wash in TBS. The slices were introduced to the DAB solution with DAB peroxidase substrate (Millipore Sigma D12384), TBS, and 30% hydrogen peroxide for 6 min. The tissue was then washed in TBS and then in 0.1 m phosphate buffer (PB), for 6 min each. Sections were mounted on glass slides using 0.05 m PB and placed in the dessicator for 25 min. The dehydration was continued through an alcohol series finishing in xylene. Mounting media (Electron Microscopy Sciences Fluoro gel with DABCO) was used to coverslip the slide. A bright-field microscope (Leica Microsystems DMR-HC) was used at 5× magnification with the same light and acquisition conditions to visualize and image each section.

### Statistical analyses

All eight mice (two WT male, two WT female, three GRov male, and one GRov female) exhibiting fluorescence with dynamic activity and identifiable cells were included in the experiment. In total, activity from 260 neurons were identified for WT and 1099 for GRov. All the neurons from each genotype were analyzed together, rather than averaging across animals, to allow identification in behavior sensitivities at the single-neuron level, as is customary for these types of neural recording data (Anacker et al., 2018; Hainmueller and Bartos, 2018). The fraction of CA1 neurons showing sensitivity to mobility or center location was compared using Fisher’s exact test as well as χ^2^ test because of sample size difference between genotypes. Pearson correlation was used to examine the relationship between the number of identified neurons and the proportion of center location-sensitive cells, and the time spent in center and the proportion of center location-sensitive cells, on a per-animal basis. Statistical analyses were performed using Python. Figures were created by Python and R and formatted in Adobe photoshop (22.5.1).

## Results

We recorded activity from 1359 dorsal CA1 pyramidal cells in four WT and four GRov mice during exploration of a novel open field to understand differences in the sensitivity of these neurons to location and locomotor behavior in a novel environment (Fig. 1*A–C*). In this group of four animals per genotype, there was no statistically significant difference in behavior in the open field, including the amount of time spent in the center of the open field (*p* = 0.77) and average velocity (*p* = 0.35; Fig. 1*D*). Velocity for all mice was on average higher in the center than in the periphery (*p* = 0.015). Immunolabeling for GR in fixed sections from these mice confirmed GR overexpression in morphologic pyramidal neurons, including in the hippocampal principal cell layers and overlying cortex (Fig. 1*E*).

**Figure 2.**
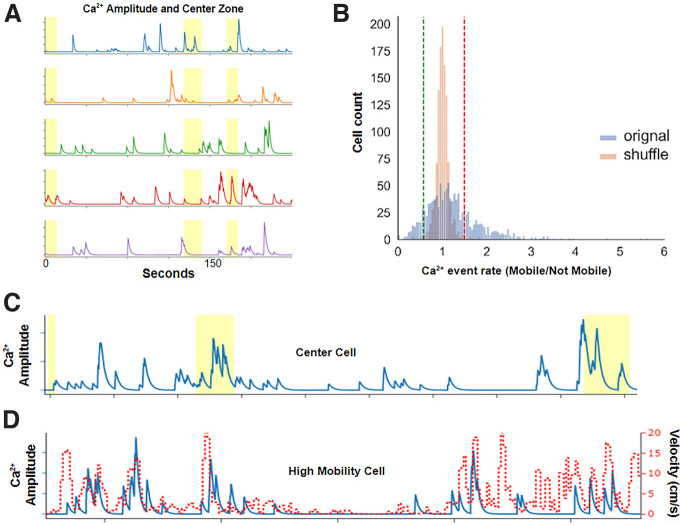
***A***, Example traces from five cells in one mouse during open field exploration. Yellow areas show when the mouse was in the center of the open field. ***B***, Example distributions of the ratio of calcium event rate in high/low mobility behavior bins for each neuron in original and shuffled data. Vertical dotted lines show thresholds for 1% (green line) and 99% (red line) of the distribution based on the shuffled data within genotype. ***C***, ***D***, Example traces of one center cell and one high mobility cell. Red line shows animal velocity (cm/s), while the highlighted yellow region shows when the animal was in the center. Blue line shows the calcium amplitude.

Dorsal CA1 neurons are expected to show sensitivity to the mobility state of the animal and the center/periphery location in the open field based on previous observations (Iwase et al., 2020; Jimenez et al., 2018). We developed a method to identify mobility-sensitive and center-sensitive cells based on a shuffled data distribution, where cells with calcium amplitude or event rate activity patterns outside the 1st or 99th percentile based on mobility or location were considered sensitive (Fig. 2*B*). Figure 2*C*,*D* illustrates the success of this method in identifying behavior-sensitive cells. We classified mobility-sensitive cells as “high mobility” or “low mobility” cells based on whether their activity increased or decreased with high mobility, respectively. We classified center location-sensitive cells as “center” or “periphery” cells based on whether their activity increased or decreased in the center, respectively.

**Figure 3.**
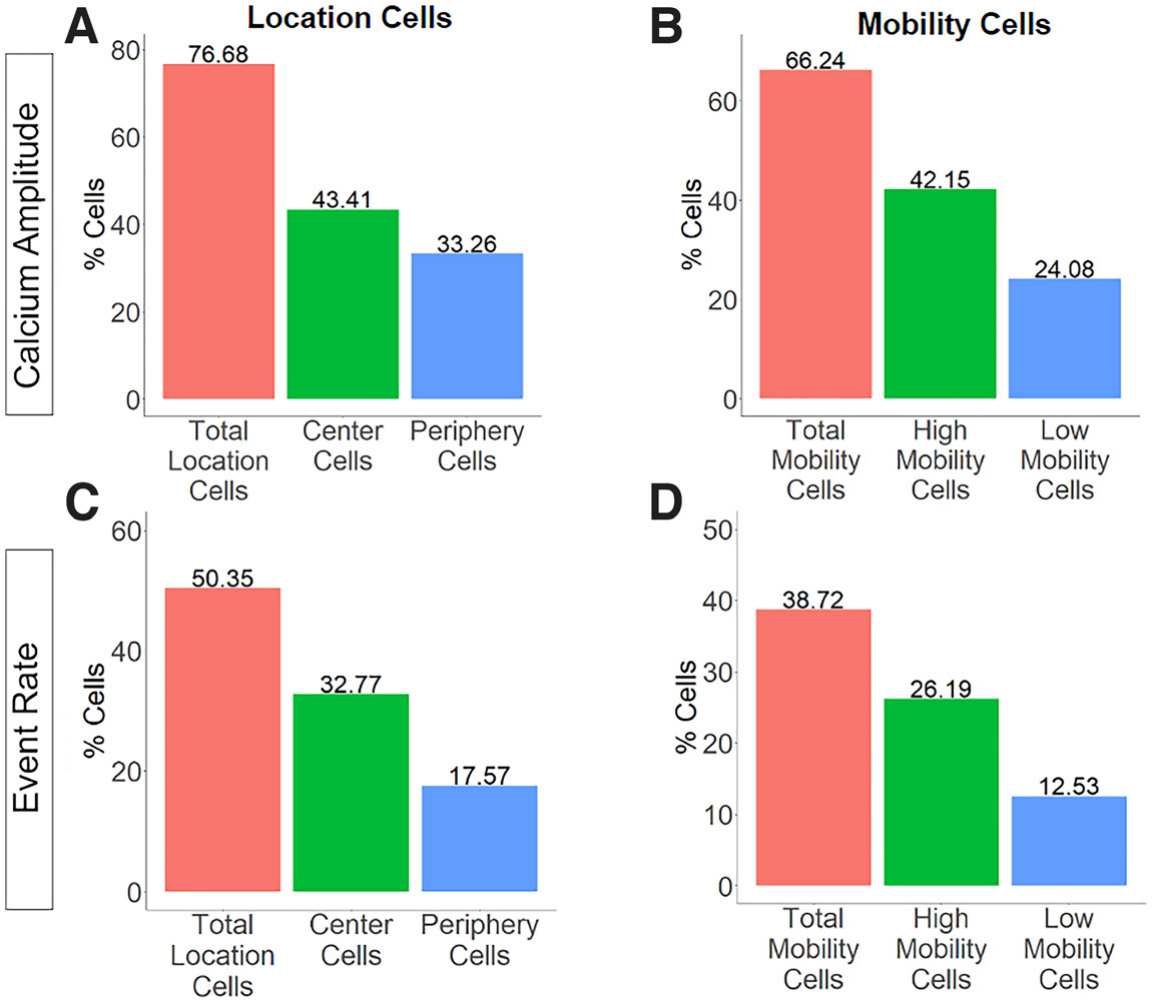
Bar graphs show the fraction of CA1 pyramidal cells out of total registered CA1 pyramidal cells (*N* = 1359) sensitive to center location (***A***, ***C***) and mobility (***B***, ***D***) based on calcium amplitude measure (***A***, ***B***) or event rate measure (***C***, ***D***).

In the analysis based on calcium amplitude, most dorsal CA1 pyramidal cells were sensitive to mobility in the open field, and the same was true for center location (Fig. 3). Consistent with prior observations, mobility-sensitive cells were more often high mobility cells than low mobility cells (Iwase et al., 2020). Center location-sensitive cells were more often center cells than periphery cells. More cells met criteria for behavior sensitivity using the calcium amplitude than the event rate measure, and all the behavior-sensitive cells identified in the event rate analysis were also identified as behavior-sensitive cells in the calcium amplitude analysis. Therefore, the calcium amplitude and event rate analyses capture similar information about behavior sensitivity, with event rate being a more stringent measure. As individual animals spent varying amounts of time in the center of the open field, we performed a correlation of the percentage of center location-sensitive cells with the amount of time spent in the center on a per-animal basis to ensure that this did not influence the results, and found no relationship (*r* = −0.1122, *p* = 0.79136).

**Figure 4.**
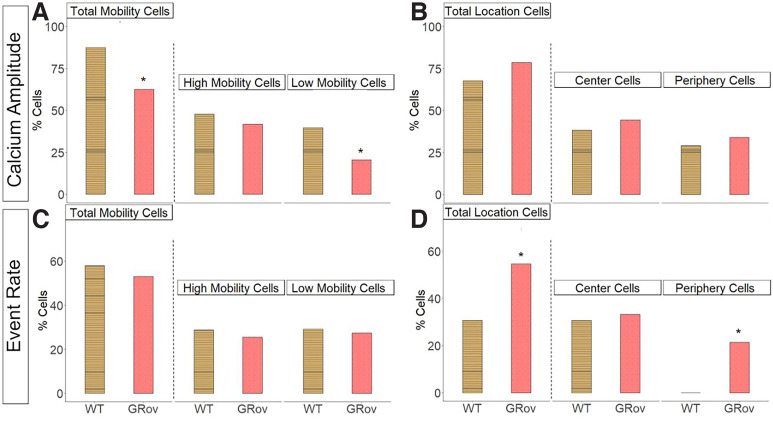
Graph shows the percentage of mobility-sensitive and location-sensitive neurons based on calcium amplitude (***A***, ***B***) and event rate (***C***, ***D***). ***A***, ***C***, Total number of mobility-sensitive cells (“total mobility cells”) based on each measure, and then these populations are broken down into high and low mobility cells. ***B***, ***D***, Total number of center-sensitive cells (“total location cells”), and then these populations are broken down into center and periphery cells. *p < 0.05 compared with WT. Extended Data Figure 4-1 shows that the statistical results were similar when compared using χ^2^ or Fisher’s exact test.

We compared the proportion of mobility-sensitive and center location-sensitive neurons between genotypes using Fisher’s exact test (Fig. 4). More WT than GRov neurons displayed mobility sensitivity: 88% of WT, 63% of GRov neurons based on calcium amplitude (*p* = 0.001; Fig. 4*A*), and 59% of WT, 53% of GRov neurons based on event rate (*p* = 0.4542; Fig. 4*C*). The difference in mobility sensitivity based on calcium amplitude appeared driven by a decrease in low mobility cells in GRov: 40% of the neurons were low mobility cells in WT (Fig. 4*A*), compared with 21% in GRov (*p* < 0.001; Fig. 4*A*). On the other hand, more GRov than WT neurons displayed center sensitivity: 68% of WT, 79% of GRov neurons displayed center sensitivity based on calcium amplitude (*p* = 0.18; Fig. 4*B*), and 31% of WT, 54% of GRov neurons displayed center sensitivity based on calcium event rate (*p* = 0.000017; Fig. 4*D*). The difference in center sensitivity based on event rate appeared driven by an increase in periphery cells in GRov: 21% of GRov and 0% of WT neurons were periphery cells (*p* < 0.001; Fig. 4*D*). Because of the large difference in sample size (number of neurons) between the WT and GRov groups, we repeated the statistical analysis using the χ^2^ test (Extended Data Fig. 4-1), which produced the same conclusion for genotype differences (*r* = 0.28, *p* = 0.4917). To ensure that the increase in center location-sensitive neurons in GRov was not an artifact of the higher number of neurons identified in GRov mice, we performed a Pearson correlation between the number of identified neurons and the percentage of center location-sensitive neurons on a per-animal basis and found no relationship (*r* = 0.28, *p* = 0.4917).

10.1523/ENEURO.0126-22.2022.f4-1 Extended Data Figure 4-1 *p*-values from χ^2^ and Fisher’s exact statistical tests to compare percentage of mobility-sensitive and location-sensitive cells between genotypes. ***A***, Statistical tests for calcium amplitude measure. ***B***, Statistical test for event rate measure. Download Figure 4-1, EPS file.

**Figure 5.**
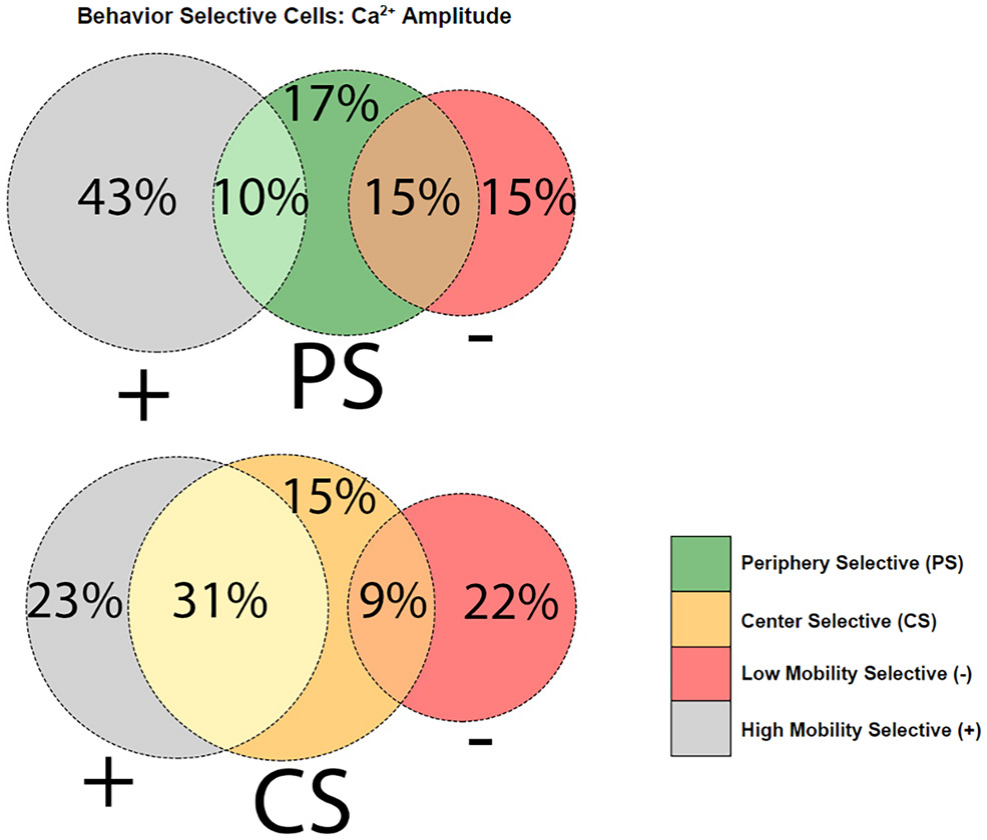
Venn diagrams show the percentage of all behavior-sensitive neurons sensitive to each behavior, with the overlap representing cells with sensitivity for both mobility and center location. + represents high mobility cells, – represents low mobility cells; CS = center cells, PS = periphery cells. Extended Data Figure 5-1 shows the overall fraction of mobility-sensitive cells calculated using only data when mice were in the periphery.

In summary, dorsal CA1 pyramidal cells in GRov showed more center sensitivity, and less mobility sensitivity, than WT neurons in the novel open field. Within the dorsal CA1 pyramidal cell population, individual cells frequently displayed sensitivity to both mobility and center location (Fig. 5). Center cells were more likely to also be high mobility cells than low mobility cells. The opposite was true for periphery cells, which were more often low mobility cells than high mobility cells (Fig. 5). Since the mice moved faster in the center of the open field (Fig. 1), this raised the possibility that the mobility analysis could be confounded by center location. We therefore repeated the mobility analysis excluding the data from the center (Extended Data Fig. 5-1). This resulted in a small decrease in the number of mobility-sensitive neurons in each category without changing the genotype pattern.

10.1523/ENEURO.0126-22.2022.f5-1 Extended Data Figure 5-1 Figure shows overall fraction of mobility-sensitive cells out of all cells only when mice were in the periphery. ***A***, ***B***, Fraction of mobility selective cells based on calcium amplitude and event rate, respectively. ***C***, Fraction of mobility-sensitive cells in WT and GRov based on calcium amplitude. Download Figure 5-1, EPS file.

**Figure 6.**
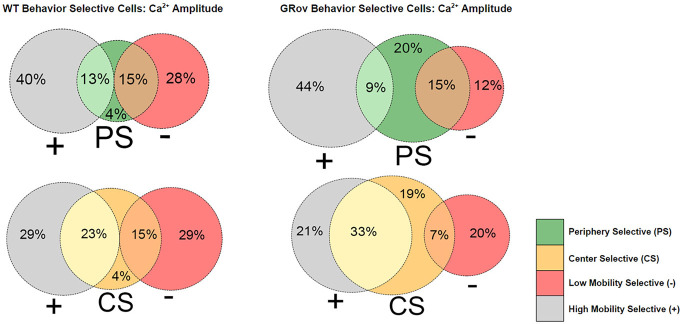
Venn diagrams for each genotype show the percentage of all behavior-sensitive neurons sensitive to each behavior, with the overlap representing cells with sensitivity for both mobility and center location, separately for neurons of each genotype. + represents high mobility cells, – represents low mobility cells; CS = center cells, PS = periphery cells.

**Figure 7.**
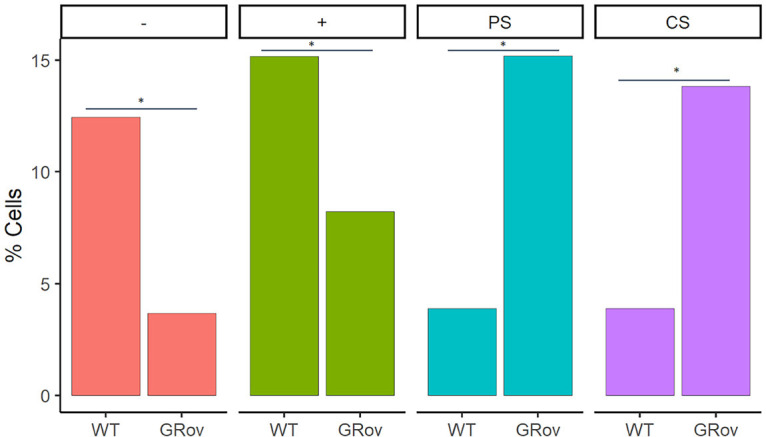
Fraction of uniquely low mobility (–), high mobility (+), center and periphery cells in WT and GRov as a percentage of the total cells. These cells showed sensitivity to either center location or mobility without overlap. *p < 0.01.

Since overlapping sensitivities were common, we sought to determine whether the GRov hippocampus had an expansion in center-sensitive cells within the mobility-sensitive population, or whether the increase in center-sensitive cells in GRov represented expansion of a uniquely center-sensitive population. Visualization of the overlap in behavior sensitivities separately for each genotype indeed suggested an expansion in the uniquely center-sensitive population of cells in GRov (Fig. 6). To quantify this, we compared the proportion of all identified neurons with unique mobility or center location sensitivities without any overlap (Fig. 7). More WT than GRov neurons showed unique mobility sensitivity: uniquely high mobility cells comprised 15% of all neurons in WT and 8% in GRov (*p* = 0.004), while uniquely low mobility cells comprised 12% of all neurons in WT and 4% in GRov (*p* < 0.001). In contrast, more GRov than WT neurons showed unique center sensitivity: uniquely center cells comprised 4% of all neurons in WT and 15% in GRov (*p* < 0.001), while uniquely periphery cells comprised 4% of all neurons in WT and 14% in GRov (*p* < 0.001). In conclusion, approximately one in three of all detected GRov neurons showed unique sensitivity to center location without any mobility sensitivity, while these unique location-sensitive cells without mobility sensitivity were rare (<1 in 10) in WT neurons.

**Figure 8.**
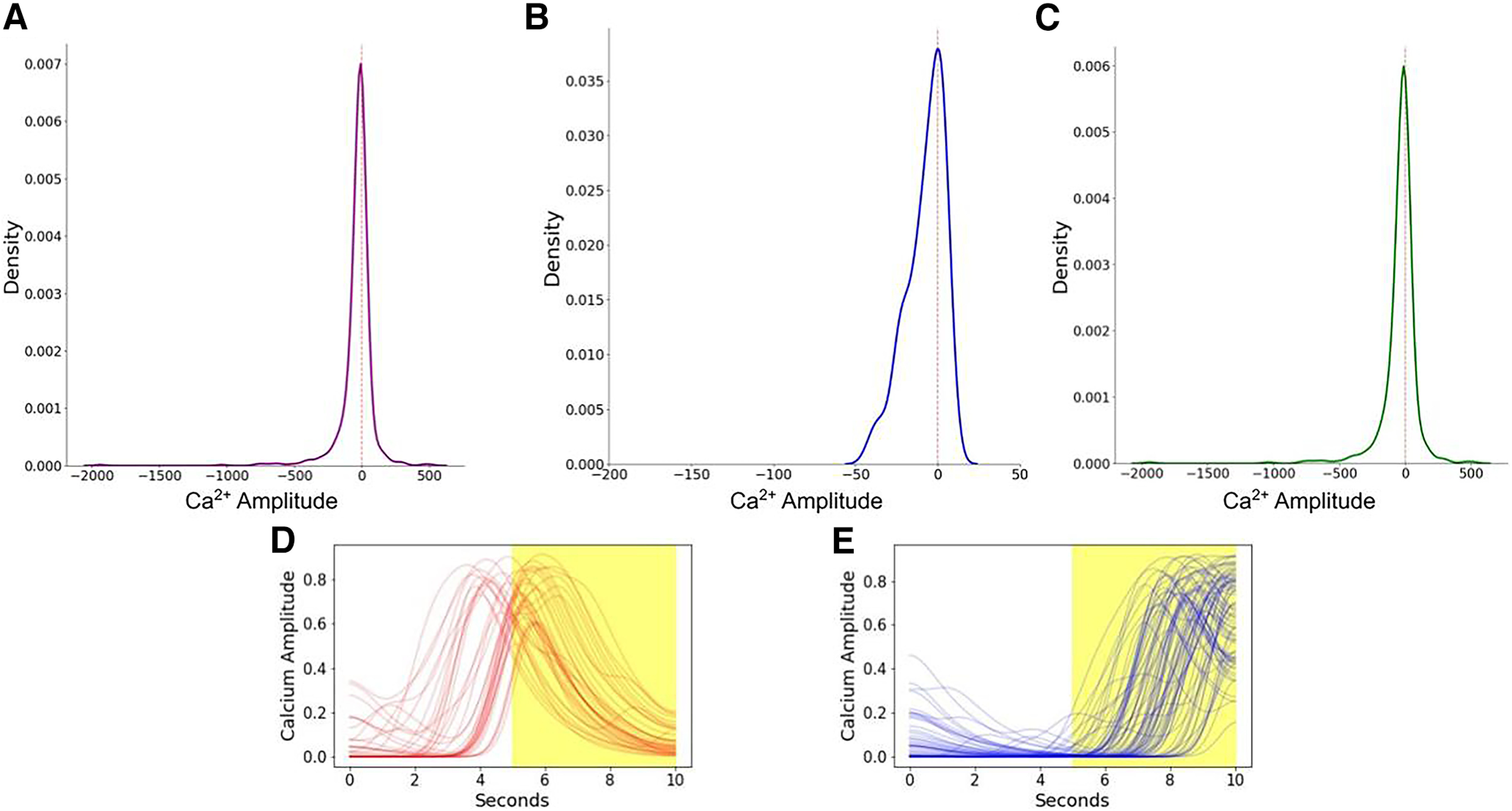
Distribution of the change in average calcium activity in the precenter bin (1 second before mouse entered into center location) from average calcium activity in the rest of the periphery bin for all center cells in the total population (***A***) and separately for WT (***B***) and GRov (***C***). Density on the *y*-axis indicates the number of cells with the given change in calcium amplitude. Most cells had a negative change in calcium amplitude, suggesting an anticipatory increase in activity just before the mouse entered the center. ***D***, ***E***, Two different neural activity patterns of center selective cells with anticipatory neural activity (***D***) and without anticipatory neural activity (***E***) when the mouse spent at least 5 s in the center.

Finally, we sought to understand whether dorsal CA1 neurons primarily represent behavior and experience, or whether they anticipate behavior and might plausibly drive it. To determine whether CA1 neuron activity might drive center exploration, we analyzed calcium activity during the 1 s before the animal entered the center and compared activity in the precenter and periphery bins in center cells (Fig. 8*A–C*). Most (69.84%) center cells showed anticipatory activity, meaning that their calcium amplitude was higher during the precenter bin in comparison to the overall periphery zone. This percentage was similar between genotypes (65% in WT, 71% in GRov). Anticipatory neurons that predict future behavior would be expected to maintain their activity during center exploration, while neurons that drive movement into the center might decay despite ongoing center exploration. We found that during periods of time when the mouse remained in the center for at least 5 s, the activity of anticipatory neurons typically decreased after center entry, while the activity of nonanticipatory neurons tended to increase (Fig. 8*D*,*E*). This pattern suggests that these anticipatory center cells could drive center exploration.

## Discussion

Here, we demonstrate that lifelong overexpression of GR in forebrain glutamatergic neurons alters the encoding of behavior-related information in a mildly aversive environment, the novel open field, by dorsal CA1 pyramidal cells. We found high rates of mobility and center location sensitivity in these neurons, with individual neurons often displaying shared sensitivities. Compared with WT, GRov neurons displayed preferential sensitivity to center location over mobility.

The high rates of mobility and location selectivity of CA1 pyramidal cells seen here are consistent with previous reports (Jimenez et al., 2018; Iwase et al., 2020). The high percentage of center-sensitive neurons makes them unlikely to be traditional position-encoding place cells, which make up a small minority of the active cells in CA1 (Hainmueller and Bartos, 2018; Stefanini et al., 2020). Our findings are novel in their demonstration of high rates of shared mobility and center sensitivities in CA1 pyramidal cells, which have not previously been compared in the same neuronal population. We further found that while individual neurons could display any pattern of shared mobility and location sensitivity, high mobility with center location and low mobility with periphery location were the most common, driven only in part by the tendency of mice to move more quickly in the center.

Between genotypes, GRov neurons displayed more center location sensitivity, and less mobility sensitivity, than WT neurons. Furthermore, more GRov than WT neurons were uniquely sensitive to center or periphery location, while more WT neurons were uniquely sensitive to mobility. Center and periphery location are often interpreted in the context of their relevance to innate anxiety states. Previously, male GRov mice showed decreased exploration of the center of an open field (Wei et al., 2004; Hebda-Bauer et al., 2010). We did not clearly see a genotype difference in overall behavior in the current study, which can be explained by many factors including the small number of mice, use of both sexes, and presence of intracranial hardware and cable tether. The lack of genotype differences in behavior overall precludes any conclusions about whether the increase in center-sensitive neurons in GRov mice drives genotype differences in behavior. We saw anticipatory activity of most center cells before center exploration. Anticipatory neuronal activity is typically seen in the setting of a predictable sequence of events, for example, with hippocampal place cells during track running or medial temporal lobe neurons during a learned sequence of images (Mehta et al., 1997; Reddy et al., 2015). In those cases, neurons begin firing in anticipation of the predicted event but maintain their firing during the event. In contrast, we found that neurons showing anticipation of center entry typically showed peak calcium activity soon thereafter followed by a decline toward baseline even when the mouse remained in the center. This pattern of neuronal activity during open field exploration suggests that these neurons specifically drive center entry, but this is a hypothesis that needs to be tested.

While our findings demonstrate heightened sensitivity to center location in an open field at the single-neuron level in dorsal hippocampus of GRov mice, the mechanism is not known. This could be a cell-autonomous effect in adult hippocampal neurons resulting from increased sensitivity to circulating glucocorticoid. In support of this possibility, prior experiments demonstrated an effect of acute and chronic stress manipulations that increase circulating glucocorticoid on hippocampal place cell function in adult mice, though the role of glucocorticoids was not tested directly (Kim et al., 2007; Park et al., 2015). Follow-up experiments should test whether glucocorticoid administration can acutely influence the behavior sensitivity of CA1 neurons. Alternatively, considering that the behavioral phenotype of the lifelong GRov mouse was recapitulated by early life GRov, the effect of GRov on CA1 function may arise from developmental cellular and circuit adaptations to GRov in early life. Additionally, as GR is overexpressed in all forebrain glutamatergic neurons, circuit adaptations may involve brain regions outside the hippocampus.

Our work has some limitations. The group sizes were unequal since more neurons were identified in GRov than in wild-type mice. This could be a biologically meaningful difference reflecting increased baseline neuronal activity, increased neuronal recruitment during open field exploration, increased pyramidal neuron density, increased susceptibility to AAV infection, or differences in calcium handling. It is also possible that this finding was because of variation in the surgical preparation (e.g., lens placement, focal plane). Based on our data, it is not possible to determine the emotional salience of the center sensitivity of dorsal CA1 neurons. Other authors have suggested that the center information encoded by dorsal CA1 neurons relates more to spatial exploration, in contrast to ventral hippocampal neurons which are proposed to encode more purely emotional information (Jimenez et al., 2018). It would be interesting in the future to determine whether this increase in the representation of center sensitivity in dorsal hippocampus in GRov also occurs in ventral hippocampus.

In conclusion, lifelong overexpression of GR in forebrain neurons alters the information encoded by CA1 pyramidal cells, leading to preferential encoding of emotionally relevant center location relative to mobility in a novel open field. We suggest that this differential encoding of experience in a novel context by dorsal CA1 pyramidal cells contributes to the differential behavioral sensitivity and more emotionally labile phenotype in GRov mice. The findings suggest that humans with developmental or lifelong differences in glucocorticoid receptor signaling may also demonstrate differential representation of experiences within the hippocampus, contributing to differential vulnerability to stress-related disorders. In the future, we recommend more consideration of the contribution of the dorsal hippocampus and its innate biases in episodic and contextual encoding in stress vulnerability.

## Synthesis

Reviewing Editor: Karyn Frick, University of Wisconsin-Milwaukee Decisions are customarily a result of the Reviewing Editor and the peer reviewers coming together and discussing their recommendations until a consensus is reached. When revisions are invited, a fact-based synthesis statement explaining their decision and outlining what is needed to prepare a revision will be listed below. The following reviewer(s) agreed to reveal their identity: Aditya Singh.

Reviewers thought that the authors had addressed many of their concerns. It was felt that the manuscript is now more focused and claims are now better balanced with the data. However, several of the most significant concerns remain, including those about sample size, large discrepancies in numbers of cells recorded per group, and statistical power.

Although an n of 4 may be a standard approach in other high impact papers, a main concern was not the n=4 per se but the large difference between the # of cells recorded in each group (260 in WT and 1099 in GRov). The reviewers thought that field of view was unlikely to explain such a large disparity in cells, and suggested that the discrepant sampling may introduce considerable statistical error towards the GRov mice.

In addition, the substantial variability evident in Fig 1D does not appear to support a correlation with the Ca2+ data, thus leading reviewers to question the conclusion that the activity of these neurons is related to open field behavior. For example, two WT mice spent less than 10 sec in the center, so it was unclear how the authors could identify cells responsive to the center when 2 of the 4 mice did not spend any time in the center. If the # of cells recorded was more similar between genotypes and/or the behavior was not as variable, the n=4 mice would not be of such concern. But given the behavioral variability and discrepancy between groups in cells recorded, the small n makes it difficult to draw any definitive conclusions. On a related note, if readers cannot safely conclude that there is a behavioral phenotype in the OFT but there is a difference in neuronal activity, then what does this mean and what are the implications of such a finding?

To strengthen the author’s interpretation, the reviewers agreed that additional animals or evidence from other approaches would be ideal. However, they also indicated that their faith in the findings would alternatively be increased if a power analysis was reported for both the WT and GRov data to ensure that statistical power is comparable across these two groups of neurons. If you choose to go this route rather than collecting additional corroborating data, then you should also add to the discussion a description of the limitations of the behavioral data (given the high variability) and specifically address how you identified neurons active in the center in the two animals that did not spend much time in the center.

Related to the time in center as a behavioral phenotype, the reviewers appreciated the anticipatory differences, however, what happens afterwards when mice cross to the center it is very important. The authors argue that this analysis cannot be conducted since mice often do not remain in the center for extended periods of time. The norm is to analyze ∼5 sec, but if mice do not stay in the center for more than 5 secs, then is there a behavioral phenotype worth investigating?

In addition to these major concerns, these more minor issues should be addressed:

--Authors may cite some additional references for anticipatory activity in hippocampal cells.

--Figure 1D should also include the bar plots with the individual data points.

--Since the study investigates neurons in dorsal hippocampus, most of the potential readers of this paper will think about place cells while reading this paper. Although place cells were measures, adding a small discussion that tries to put the findings from this study with the preexisting place cell literature (such as stress-related place cell findings) would be appreciated by the readers.

## Author Response

Response to Reviewers

EDITOR/REVIEWER COMMENT: Reviewers thought that the authors had addressed many of their concerns. It was felt that the manuscript is now more focused and claims are now better balanced with the data. However, several of the most significant concerns remain, including those about sample size, large discrepancies in numbers of cells recorded per group, and statistical power.

Although an n of 4 may be a standard approach in other high impact papers, a main concern was not the n=4 per se but the large difference between the # of cells recorded in each group (260 in WT and 1099 in GRov). The reviewers thought that field of view was unlikely to explain such a large disparity in cells, and suggested that the discrepant sampling may introduce considerable statistical error towards the GRov mice.

In addition, the substantial variability evident in Fig 1D does not appear to support a correlation with the Ca2+ data, thus leading reviewers to question the conclusion that the activity of these neurons is related to open field behavior. For example, two WT mice spent less than 10 sec in the center, so it was unclear how the authors could identify cells responsive to the center when 2 of the 4 mice did not spend any time in the center. If the # of cells recorded was more similar between genotypes and/or the behavior was not as variable, the n=4 mice would not be of such concern. But given the behavioral variability and discrepancy between groups in cells recorded, the small n makes it difficult to draw any definitive conclusions. On a related note, if readers cannot safely conclude that there is a behavioral phenotype in the OFT but there is a difference in neuronal activity, then what does this mean and what are the implications of such a finding?

AUTHOR RESPONSE:

We thank the reviewers for their favorable remarks about the first revision and for their additional comments and suggestions.

We understood the reviewers were concerned that the total number of cells identified per animal might bias the results if the number of identified cells relates in any way to the proportion of center location cells, since more cells were identified in Grov mice than in WT mice. To address this point we performed a correlation analysis to determine whether the number of cells was related to the proportion of center location-sensitive cells on a per-animal basis. Pearson correlation revealed no significant correlation between the total number of registered cells and the fraction of center location-sensitive cells (r=0.28, p=0.4917).

We also understood the reviewers were concerned that the amount of time animals spent in the center might affect the ability to identify center location-sensitive cells, biasing the results. This is important considering the large variation in behavior between mice, which we note is typical for mouse open field behavior. To address this, we performed a correlation between the time spent in the center and fraction of location selective cells on a per-animal basis, and found no relationship (r=-0.1122, P=0.79136).

The results of these two new correlation analyses are reported in the Results section of the revised manuscript (lines 190-194 and lines 209-213).

EDITOR/REVIEWER COMMENT: To strengthen the author’s interpretation, the reviewers agreed that additional animals or evidence from other approaches would be ideal. However, they also indicated that their faith in the findings would alternatively be increased if a power analysis was reported for both the WT and GRov data to ensure that statistical power is comparable across these two groups of neurons. If you choose to go this route rather than collecting additional corroborating data, then you should also add to the discussion a description of the limitations of the behavioral data (given the high variability) and specifically address how you identified neurons active in the center in the two animals that did not spend much time in the center.

AUTHOR RESPONSE:

The method for identifying center location-sensitive cells was the same for all neurons regardless of the total amount of center time per neuron/animal.

The statistical conclusions in this study are the same regardless of whether the Fisher’s exact test or Chi squared test is used to compare the proportions, and we have added this to the revised manuscript (lines 207-209). To address the reviewers’ concerns about power, The Table S1 below provides power analyses for the analysis comparing the proportion of center location and mobility sensitive cells between WT and GRov mice based on calcium amplitude (Table S1- A) and event rate (Table S1-B). The data showed power of 1 where the genotype comparisons were statistically significant ((Table S1-A) Calcium amplitude; total mobility cells, low mobility cell. (Table S1-B) Event rate; total location cells, periphery cells). While this power analysis was based on the Chi squared test, it is expected to be similar for the Fisher’s exact test, where the statistical conclusions were the same. We chose not to add the power analysis to the manuscript.

TABLE S1-A

WT

(N = 260)

GRov

(N = 1099)

Power P value

(WT vs GRov)

Pr (Total Mobility Cells) 0.8753 0.6258 1 0.0012 *

Pr (Low Mobility

Cells)

0.3968 0.2066 1 0.0000020*

Pr (High Mobility

Cells)

0.4785 0.4192 0.4122 0.3069

Pr (Total Location

Cells)

0.6770 0.7864 0.9509 0.1801

Pr (Center Cells) 0.3852 0.4449 0.4144 0.4449

Pr (Periphery Cells)

0.2917 0.3415 0.3306 0.3037

TABLE S1-B

WT

(N = 260)

GRov

(N = 1099)

Power P value

(WT vs GRov)

Pr (Total Mobility Cells) 0.5797 0.5310 0.2922 0.4542

Pr (Low Mobility

cells)

0.2918 0.2749 0.0811 0.4531

Pr (High Mobility

cells )

0.2879 0.2561 0.1863 0.7131

Pr (Total Location

Cells)

0.3073 0.5464 1 0.000017*

Pr (Center cells) 0.3073 0.3321 0.1133 0.6255

Pr (Periphery cells)

0 0.2143 1 <0.005*

EDITOR/REVIEWER COMMENT: Related to the time in center as a behavioral phenotype, the reviewers appreciated the anticipatory differences, however, what happens afterwards when mice cross to the center it is very important. The authors argue that this analysis cannot be conducted since mice often do not remain in the center for extended periods of time. The norm is to analyze ∼5 sec, but if mice do not stay in the center for more than 5 secs, then is there a behavioral phenotype worth investigating?

AUTHOR RESPONSE:

We thank the reviewer for this comment. We have added Figure 8D-E exploring the typical pattern for anticipatory and non-anticipatory cells when the mouse remained in the center for at least 5 seconds. There we show a typical pattern for “anticipatory” cells to peak in their activity just before or after center entry and then decline. While this suggests that these cells could plausibly drive the behavior of center entry, we agree with the reviewer that the lack of genotype differences in behavior in this case prevents us from drawing this conclusion, and this limitation is addressed in the Discussion (lines 276-281).

EDITOR/REVIEWER COMMENT: In addition to these major concerns, these more minor issues should be addressed:

--Authors may cite some additional references for anticipatory activity in hippocampal cells.

AUTHOR RESPONSE: These were added to the Discussion (Line 284).

EDITOR/REVIEWER COMMENT: --Figure 1D should also include the bar plots with the individual data points.

AUTHOR RESPONSE: The goal of this study was not to compare behavior between genotypes, and the small number of animals prevents any definitive conclusions on that point. For this reason, we chose not to add summary data (bar plots) to the behavior graphs.

EDITOR/REVIEWER COMMENT: --Since the study investigates neurons in dorsal hippocampus, most of the potential readers of this paper will think about place cells while reading this paper. Although place cells were measures, adding a small discussion that tries to put the findings from this study with the preexisting place cell literature (such as stress-related place cell findings) would be appreciated by the readers.

AUTHOR RESPONSE: We added this information to the revised manuscript (Line 297).
